# Modelling land use-induced foraging distributions of flying foxes and emerging spillover risks

**DOI:** 10.1016/j.onehlt.2026.101333

**Published:** 2026-01-14

**Authors:** Erin Stafford, Åke Brännström, Kyrre Kausrud, Henrik Sjödin

**Affiliations:** a Department of Epidemiology and Global Health, Umeå University, Umeå, Sweden; b Department of Mathematics and Mathematical Statistics, Umeå University, Umeå, Sweden; c Complexity Science and Evolution Unit, Okinawa Institute of Science and Technology Graduate University (OIST), Okinawa, Japan; d Advancing Systems Analysis Program, International Institute for Applied Systems Analysis (IIASA), Laxenburg, Austria; e Norwegian Veterinary Institute, Ås, Norway; f Department of Wildlife, Fish and Environmental Studies, Swedish University of Agricultural Sciences, Umeå, Sweden

**Keywords:** Mathematical modelling, Flying foxes, Optimal foraging theory, Land-use and land-cover change, Zoonotic spillover

## Abstract

Despite their critical role as reservoir hosts for many zoonotic diseases, the impact of land-use and land-cover changes (LCLUC) on flying foxes' interactions with humans remains unclear, posing a potential public health risk. To address this, we apply optimal foraging theory and individual-based modelling to simulate flying-fox movement and population dynamics under various LCLUC scenarios. After validating our model against available data, we analyze the effects of agriculturalization, urbanization, forest fragmentation, and reforestation on flying-fox densities across synthetic landscapes of urban, forest, orchard, and water-body habitats. Our findings indicate that habitat disruption—particularly fragmentation through urbanization—significantly increases the risk of zoonotic spillover events by increasing contacts between species. Scenarios of forest degradation reveal that ecologically degraded forest environments can further exacerbate this risk. Additionally, we find that reforestation can alleviate spillover risk. These results underscore the importance of conservation and habitat restoration as critical strategies for mitigating zoonotic disease transmission.

## Highlights

• Individual-based model links land-use to zoonotic spillover risk from flying foxes.
• Urbanization-driven fragmentation produces the largest increase in spillover risk.
• Reforestation, especially of agricultural areas, most effectively reduces risk.
• Forest quality (resource-rich vs. poor) mediates how urbanization affects spillover.
• Model reveals how landscape configuration shapes human–bat contact intensity.

## 1 Introduction

The majority of emerging infectious diseases (EIDs) can be characterized as zoonotic diseases [1], [2]. These diseases, which typically spread between vertebrate animals and humans, account for over 60% of all human pathogens [1], [3], [4]. Due to factors like climate change [5], [6], [7], land-use change [5], [8], [9], [10], [11], [12], and global travel [3], [4], zoonotic diseases pose an increasing threat to public health [1]. Land-cover and land-use changes (LCLUC), including deforestation, fragmentation, agricultural expansion, and urbanization, alter the risk of zoonotic spillover by altering vector and host community composition, human and animal behavior, the spatial distribution of populations, and ecosystem biodiversity [3], [4], [8], [13]. As over 70% of emerging zoonotic diseases originate in wildlife [1]—a consequence of pathogen dependence on living hosts rather than any inherent risk of wildlife—understanding the effects of environmental disturbances and changes on reservoir species populations in wildlife is crucial for evaluating the risk of zoonotic disease transmission. Moreover, because biodiversity loss can amplify these effects [14], [15], there is potential to develop synergetic land-management solutions that promote ecosystem stability and mitigate disease risk.

Flying foxes (genus *Pteropus*), a taxonomic group of Old-World fruit bats (family *Pteropodidae*) native to Africa, Asia, and Australia [11], are believed to be particularly good reservoir hosts [16]. This is due to a few key traits: roosting and migratory behaviors, which facilitate disease spread; hibernation and torpor, which allow outbreaks to persist across seasons; extreme longevity, which enables long-term transmission; and unique immune responses, which reduce disease severity [17]. Notably, flying foxes transmit Nipah and Hendra viruses [11], [18], [19], [20], [21], [22]. Both cause severe encephalitis, with case-fatality rates of 40–75% and ~ 57%, respectively [23], [24], [25]. They can also spread Ebola virus [26], [27], [28], Australian bat lyssavirus [29], [30], coronaviruses [31], and others [16].

The migratory and foraging behaviors of flying foxes, that may drive zoonotic spillover, also render the flying foxes indispensable to the ecosystems they inhabit. By serving as essential pollinators and seed dispersers for tree species bearing large fruit and seeds [32], [33], flying-fox populations are crucial for maintaining natural forest structure and promoting forest regeneration—processes that contribute to ecosystem resilience and support healthy ecological communities of diverse wildlife species [34]. High species diversity within ecosystems has a dilution effect on pathogen transmission, as having abundant non-host species reduces the likelihood of infection in the more competent hosts—acting as a natural barrier to disease emergence [12], [14], [15]. Therefore, it is important to develop mitigation strategies that integrate sustainable development with habitat preservation. Such approaches reinforce ecological resilience—by maintaining landscape heterogeneity and conserving flying-fox populations—and help to lessen zoonotic spillover risk [33], [35], [36].

The increasing threat posed by flying fox-borne diseases is closely linked to LCLUC [11], yet the specific mechanisms by which different LCLUC scenarios alter the spatial density and distribution of these reservoir hosts remain poorly understood [11], [37], [38], [39]. Targeted research—especially into how specific LCLUC scenarios (agricultural expansion, urbanization, fragmentation) affect flying-fox population densities and spatial distributions—is urgently needed to inform land-management interventions that both mitigate spillover and conserve ecosystem services [38], [40].

Recent research has attempted to bridge the knowledge gap. Accounting mainly for large-scale mobility patterns, studies conducted in Australia have shown that land-use change has increased flying fox presence in agricultural areas and that food shortages have contributed to a rise in spillover events [10], [41]. Empirical studies on large-scale movements of flying foxes (*Pteropus* spp.) have mostly used satellite telemetry to track movements across multiple countries [42], [43], [44]. In contrast, the smaller-scale foraging patterns of these animals have been analyzed through GPS tracking [46], [47]. However, the results of these studies are inconsistent or incomplete due to differences in landscapes and uncertainties arising from small sample sizes, often involving only a few GPS-tracked individuals. In conclusion, empirical studies on important LCLUC effects in relation to both small- and large-scale mobility patterns of flying foxes are limited. The complexity of these processes together with the logistics demanded on the required spatiotemporal scales make empirical studies inherently challenging. Under such conditions, mathematical modelling is often an advantageous complement that can efficiently outline general principles and processes and help bridge knowledge gaps. Yet, to our knowledge, only one study has applied mathematical modelling to flying fox movement in the context of large-scale migration [45], and none have described or predicted small-scale foraging movements.

Here, we present the first mathematical model that captures the small-scale movements of flying foxes. Our model reduces their complex daily behaviors to three principal components: roosting, travelling, and foraging. These three components are explainable by modelling and represent the majority of daytime activities (roosting) [48] and nighttime behaviors (travelling and foraging) [46], [47]. The model, which is individual-based and grounded in optimal foraging theory, is presented in Section 2. Using this model, we simulate and study the small-scale geographic movements of flying foxes under various LCLUC and forest degradation scenarios. Based on these simulations, we estimate potential changes in zoonotic spillover risk and present our findings in Section 3. Finally, in Section 4, we summarize our findings and contextualize our results within the One Health framework, highlighting key insights and broader implications.

## 2 Methods

**Fig. 1:**
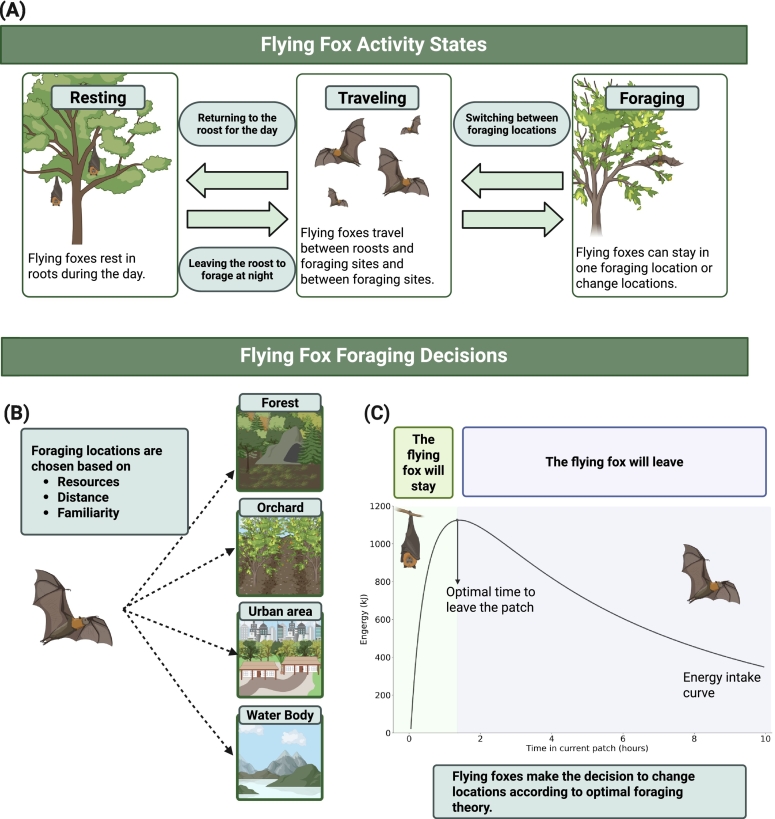
Visualization of flying fox individual-based processes. Panel (A) shows the daily activities included in the flying-fox model: resting, travelling, and foraging. Panel (B) shows which types of patches flying foxes can choose to forage in as well as the factors that influence these decisions. Panel (C) shows how optimal foraging theory is used in the individual-based model. Foraging in a patch sees diminishing returns as the patch resources are consumed, so flying foxes optimize their net energy intake by leaving when the travel time to the next patch balances the average gain in intake rate from finding a new patch. (Created in BioRender. Stafford, E. (2025) https://BioRender.com/uu287h9).

**Fig. 2:**
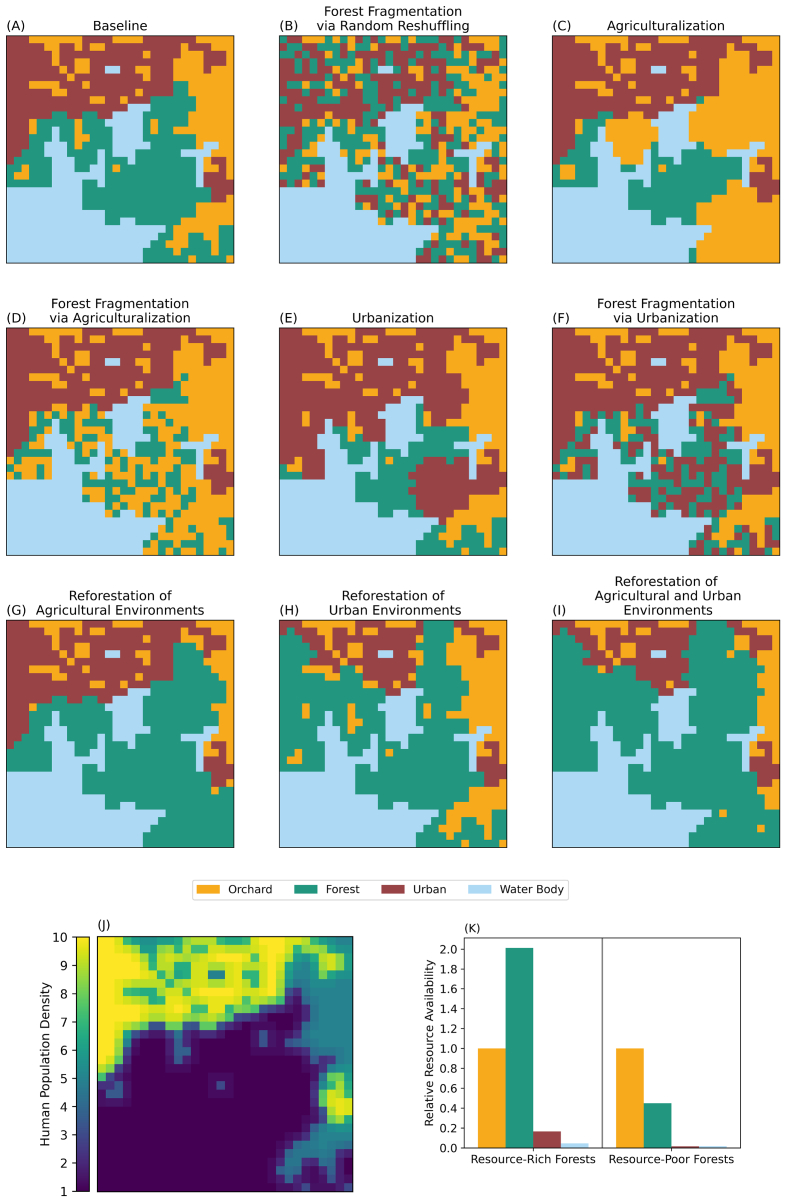
Visual description of methods used for simulating synthetic landscapes. Panels (B)-(I) show examples of landscape changes from the baseline (A). Panels (B)-(F) show examples where 50% of the forest patches have either been switched with or converted to other patch types. Panels (G) and (H) show examples where 50% of the urban or orchard patches have been converted to forest patches, and Panel (I) shows an example where 50% of urban patches and 50% of orchard patches have been converted to forest patches. Panel (J) shows an example of the human population distribution for the baseline scenario in Panel (A), illustrating that humans are most dense in urban patches and least dense in water body and forest patches. Panel (J) also visualizes edge effects arising from the dispersion of human populations from high-density urban patches into adjacent lower-density landscape types, which alters average population sizes in boundary zones. Panel (K) shows the average resource distribution of each patch type relative to the orchard patches when keeping the total amount of resources fixed. The total amount of resources is fixed in order to explore the effects of landscape structure alone on bat populations, which would not be possible in traditional ecological studies. Created in BioRender. Stafford, E. (2026) https://BioRender.com/i7lodx9.

We developed a spatially explicit individual-based model (IBM) to evaluate how land-cover and land-use change (LCLUC) influences flying-fox foraging behavior and associated zoonotic spillover risk. The model follows the ODD (Overview, Design concepts, and Details) protocol for individual-based models [49]. Descriptions of the IBM, LCLUC implementation, and spillover risk calculation are provided below. A schematic representation of the model structure and processes is shown in Fig. 1, and the representative LCLUC configurations used in simulations are illustrated in Fig. 2.

### 2.1 Overview of IBM

Two agent types are represented in the model: flying foxes and landscape patches. Individual flying foxes alternate between roosting (day), travelling, and foraging (night). The landscape comprises a 30 × 30 grid of 1 km^2^ patches classified as forest, orchard, urban, or water (baseline proportions: 30%, 20%, 25%, 25%), each with regenerating resources.

### 2.2 Design concepts of the IBM

Agents make movement and patch-leaving decisions that maximize net energy gain under optimal foraging theory, balancing intake against travel, metabolic costs, and within-patch diminishing returns. Optimal foraging theory (OFT) provides a mechanistic link between behavioral decisions and energetic constraints, positing that animals maximize net energy gain by trading off intake against search and movement costs [50], [51], [52], [53], [54], [55].

In movement ecology, OFT has strongly influenced agent-based models that treat organisms as energy-limited, utility-based agents whose movement and patch-leaving rules account for time and energy budgets [50], [56]. Previous applications have included grazing ungulates [57], [58], birds [59], [60], fish [61], [62], and primates [63], incorporating factors like energetic considerations in group behavior. While not all models implement the marginal value theorem explicitly, their decision rules are OFT-compatible, as individuals compare expected gains with movement and metabolic costs to decide when to remain or switch patches.

Following this logic—similar to the approach in [45]—individual flying foxes in our model behave in ways that statistically maximize net energy gain, accounting for metabolic costs, resource competition, and the need for dietary diversity. An individual leaves a foraging patch when marginal energy gain falls below the average gain rate (Fig. 1C) and switches roosts when the expected nightly energy gain from an alternative roost exceeds that of the current one. Moreover, roost-switching in this framework is restricted to a local-to-regional spatial extent and represents short-range relocation among nearby roosts that are assumed to be suitable, rather than large-scale seasonal migration. Fine-scale roost attributes and environmental drivers are instead captured at the patch level through energetic trade-offs. Patch-leaving is deterministic, while patch selection is stochastic, governed by distance, familiarity, and resource availability (Fig. 1B). Further details of the mathematical model are provided in the Appendix and in Fig. S1 in the Supplementary Information.

### 2.3 Details of the IBM

Initialization includes two roosts located in forest patches (when available), consistent with observed roost-switching behavior [64]. Individuals (*n* = 10,000) are randomly assigned initial foraging histories and roost locations. Each simulation spans 90 days within a single season with constant population size. While the model does not distinguish among specific seasons, this interval is intended to represent a period when food resources are available in both orchard and forest habitats (i.e., a fruiting period) and is assumed to occur outside of breeding periods, which could otherwise affect foraging behavior. Resource capacities and regeneration rates vary among patch types, but total resource availability across the 30 × 30 grid is held constant across scenarios to isolate structural effects.

The model was parameterized using published data on the metabolic rates and foraging behaviors of flying foxes in Southeast Asia, where bat-borne zoonoses are an increasing threat [23], [24], [25]. Parameter values were drawn primarily from studies of the grey-headed flying fox (*Pteropus poliocephalus*), the most comprehensively studied species [65], [66]. Parameters lacking empirical estimates were tuned to reproduce realistic energy balance and movement dynamics. Calibration on a synthetic test grid targeted two criteria: (i) stable energetic equilibrium and (ii) two to three foraging-site changes per night.

We validated model outputs against GPS-tracking studies of flying-fox foraging and movements, focusing on two Southeast Asian datasets [46], [47], which are closest to our geographical areas of interest. The first of these studies followed eight individuals roosting in a Buddhist pagoda in Kandal Province, Cambodia, during April–May 2016 [46], while the second tracked nineteen individuals from multiple temple roosts in central and eastern Thailand [47]. Despite the context-specific variability in GPS data [46], [47], [67], our simulated nightly travel distances (mean ≈ 30 km) and habitat-use patterns—showing predominant foraging in orchards and other productive, human-modified habitats—closely matched empirical observations once land-cover classifications were harmonized. The number of foraging trips per night also aligned with reported values (< 2 per night). Together, these comparisons demonstrate that the model captures key behavioral mechanisms of *Pteropus* spp. relevant to understanding how resource distribution and landscape change shape spillover risk. Additional information on fitting and validation is provided in the Appendix.

### 2.4 Implementation of land-cover/land-use change (LCLUC) scenarios

Land-cover/land-use change (LCLUC) scenarios include agricultural expansion, urban expansion, forest fragmentation (via agriculturalization, via urbanization, or via random reshuffling), and reforestation (urban, orchard, or both). LCLUC scenarios are implemented through replacing forest patches with either urban or orchard patches (Fig. 2A-I). In expansion scenarios, forest patches that are adjacent to the anthropogenic landscapes are replaced. To implement fragmentation, random forest patches are selected for replacement. Forest resource abundance is varied to represent resource-poor vs resource-rich conditions (Fig. 2K).

### 2.5 Calculation of spillover risk

Spillover risk was computed as the probability of one or more spillover events per simulation based on the contact-adjusted force of infection. Potential contacts were estimated using the average bat activity in each patch, assuming human density was highest in urban patches, moderate in orchards, and lowest in forests and water bodies. Increased human activity in zones adjacent to urban and agricultural patches captured edge effects that can strongly influence contact rates [68]. Further details of the spillover-risk computation and visualization of edge effects are provided in the Appendix (Section A.2) and in Fig. 2J, respectively.

## 3 Results

We find that the land use-induced foraging distributions of flying foxes in each of the eight modelled LCLUC scenarios (Fig. 2) greatly impacts the risk of zoonotic spillover, by either increasing spillover risk through increasing anthropogenic land-use, or by reducing spillover risk through natural habitat-regeneration, i.e. reforestation. Additionally, we find that forest degradation, represented by resource-poor forests, worsens spillover risk for most LCLUC scenarios. We present more on these results in the following subsections.

### 3.1 Land-cover and land-use change (LCLUC) impacts the risk of zoonotic spillover

**Fig. 3:**
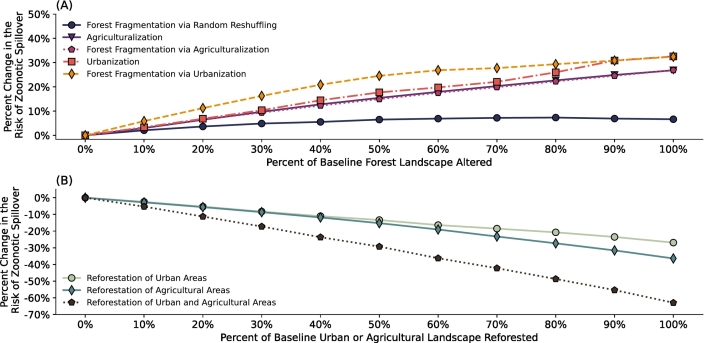
Change in the risk of zoonotic spillover from LCLUC scenarios relative to the baseline scenario. Panel (A) shows that LCLUC scenarios that reduce forest sizes and continuous coverage increase the risk of zoonotic spillover from the baseline scenario. Forest fragmentation via urbanization has the greatest effect, the effects of agriculturalization, fragmentation via agriculturalization, and urbanization were similar, and forest fragmentation via random reshuffling had only a small effect. Panel (B) shows that LCLUC scenarios that increase forest sizes has the inverse effect with reforesting agricultural areas having a greater impact than reforesting urban areas.

Forest fragmentation via urbanization had the greatest effect across all degrees of forest loss (Fig. 3A). For instance, at a 50% forest-to-urban conversion, the risk of zoonotic spillover is approximately 25% higher compared to the baseline scenario (i.e., 0% conversion), and at 100% forest-to-urban conversion the increase in the risk of zoonotic spillover is approaching approximately 33%. This large impact is due to the compounded effects of urbanization and fragmentation in this LCLUC scenario. This is because increased urbanization—whether through forest fragmentation or direct expansion into forests—reduces natural habitat and forces flying foxes to congregate in resource-rich orchards (see Section 3.3). These areas have higher human population densities than forests, resulting in elevated contact rates between flying foxes and humans. Fragmentation increases the risk of zoonotic spillover by expanding forest–urban border areas, which amplifies edge effects. In the model, edge effects arise primarily because humans at these interfaces are assumed to frequently cross between densely populated urban areas and adjacent forest patches. Flying fox use of neighboring urban patches can also occur as a consequence of the model dynamics, with inter-patch distance influencing patch selection. Under the current parameterization, such movements are rare due to low resource availability in urban areas. Higher resource availability would naturally increase bat use of these patches and the associated opportunities for contact.

Urbanization via direct expansion into forests, having similar effects as fragmentation via urbanization, had the second greatest effect across all degrees of forest loss, and leads to an approximately 18% increase in risk when 50% of the forest habitat is urbanized (Fig. 3A).

Agriculturalization, via direct expansion into forests and via forest fragmentation, had the third greatest effect across all degrees of forest loss as each produced an ~15% increase in spillover risk when 50% of forest was converted (Fig. 3A). Because bats already spend the majority of their foraging time in orchards in the baseline, converting forest to agriculture induces little additional behavioral shift—foraging effort was already concentrated in high-resource, higher-human-density orchard patches. As a result, both agriculturalization pathways yield similar risk increases, driven mainly by forest loss rather than substantial reallocation of foraging (see Fig. S1 in the Supplementary Information for a visualization of behavior changes). This suggests that limiting overall forest loss is more consequential than the specific mechanism of agriculturalization.

Forest fragmentation via random reshuffling had a much less significant effect than the other LCLUC scenarios that fragment or reduce the forested landscape (Fig. 3A). When 50% of the forested landscape was altered, there was only a ~ 6% increase in the risk of a zoonotic spillover event occurring. This is because, in this scenario, the proportion of the different patch types in the larger landscape doesn't change, only the size of the forest clusters is reduced. As the grid is small enough for distances between patches to only have a small effect on patch choice, the proportion of time that flying foxes spent in each patch type wasn't changed. The increase in the probability of zoonotic spillover is solely due to the edge effects at the urban-forest and forest-orchard interfaces, which arise from landscape configuration rather than changes in individual behavior. In this framework, the number of bordering urban or agricultural patches reflects the extent of forest–anthropogenic boundaries surrounding a forest patch: a larger number of neighboring urban or agricultural patches implies a greater total boundary length over which interactions between humans and flying foxes can occur. As shown in Fig. S2 (Supplementary Information), forest patches bordered by more urban and agricultural patches experience higher average bat–human contact rates, illustrating how increases in boundary extent alone can elevate spillover risk. Moreover, the effect of fragmentation via random reshuffling peaks at 80% of baseline forest alteration and levels off beyond that point (Fig. 3A). This is because forest clusters at this point start to reform as more of the forest patches are switched with urban or orchard patches in the reshuffling process.

### 3.2 Effects of reforestation on zoonotic spillover risk

Fig. 3B shows how reforestation scenarios affect the risk of spillover. These results show that reforestation of urban environments, orchards, and the combined reforestation of orchard and urban environments all reduce the risk of zoonotic spillover. The combined reforestation of orchard and urban environments has the largest impact, with a 29% decrease in the risk of zoonotic spillover when 50% of orchards and 50% of urban environments are reforested. This, however, represents a much more intense reforestation than the scenarios where either 50% of urban areas or 50% of orchards were reforested because many more grid patches were converted to forest patches overall. When 25% of each orchard and urban patches were reforested, the reduction in spillover risk was about 14%. This is a similar level of patch conversion to the reforestation of either 50% of urban patches or 50% of orchard patches alone. Reforestation of 50% of urban areas alone reduced spillover risk by about 13%, while reforestation of 50% of agricultural areas alone reduced spillover risk by approximately 15%. These results show us that reforestation efforts expanding forested habitat in agricultural areas would be most effective in reducing zoonotic spillover. This aligns with the results of Fig. 3A showing the impact of agricultural expansion.

### 3.3 Effects of resource availability on spillover risk under LCLUC scenarios

**Fig. 4:**
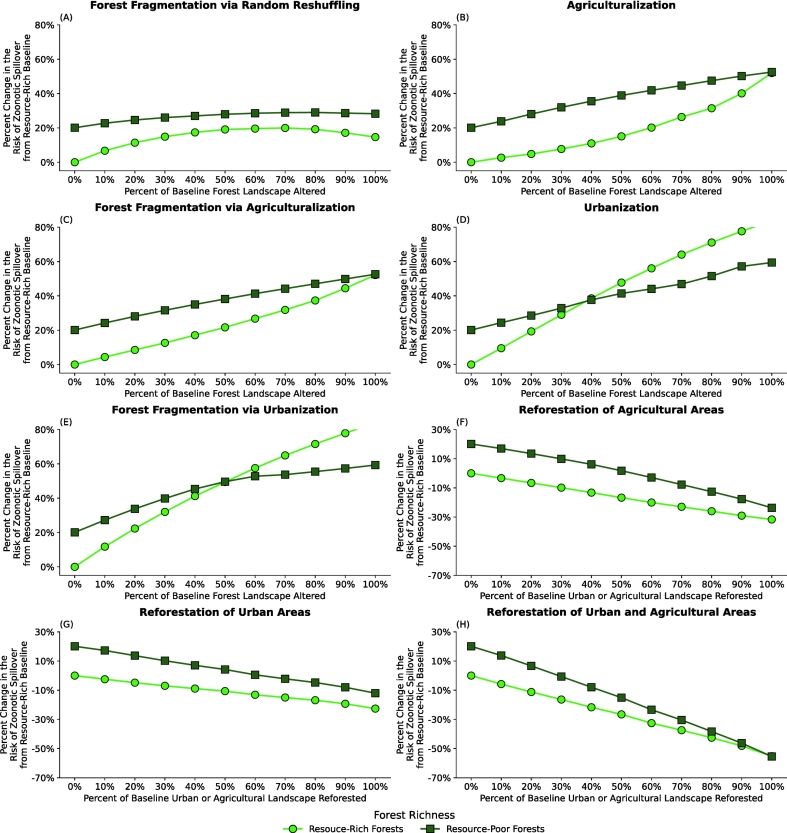
Differences in forest resource abundance led to differing responses to land-use change scenarios and, consequently, to spillover risk. Panels (A) - (H) show the effects of forest resource abundance in each of the LCLUC scenarios. Here, the plots show the relative risk of zoonotic spillover events occurring under LCLUC scenarios for resource-rich forests and resource-poor forests compared to the baseline, which is 0% change in landscape structure and resource-rich forests.

To assess how underlying resource availability modulates the effects of landscape change on spillover risk, we compared outcomes across two forest resource conditions: resource-poor and resource-rich forests (See Fig. 4). While the degree of forest resource abundance is here an accurate description in relation to our model assumption, degrees of resource abundance could be interpreted as (inversely) degrees of forest degradation (e.g., through partial logging or similar). Overall, both environments showed qualitatively similar trends—spillover risk increased with greater landscape disturbance and decreased under reforestation—yet the magnitude of these effects differed. In most scenarios, landscape change resulted in a larger percent change in risk in resource-poor forests relative to resource-rich ones (when compared to the baseline scenario with resource-rich forests). However, urbanization exhibited a distinct pattern: resource-poor forests experienced higher relative changes in risk at low to moderate levels of urban expansion (up to ~40% via direct expansion or ~ 50% via fragmentation), beyond which risk increased more steeply in resource-rich forests. This crossover reflects the interplay between habitat quality and edge effects. In degraded, resource-poor forests, flying foxes already rely heavily on orchards, leading to early aggregation in human-dominated areas. In contrast, in resource-rich forests, urbanization introduces new forest edges and fragmented forest habitats that amplify edge effects, sharply increasing contact rates. Thus, while resource scarcity amplifies spillover risk under most forms of landscape disturbance, the creation of new urban-forest interfaces in healthy forests can, beyond a certain point, become the dominant driver of elevated spillover potential.

### 3.4 Land-cover and land-use change impacts the human-animal interfaces

**Fig. 5:**
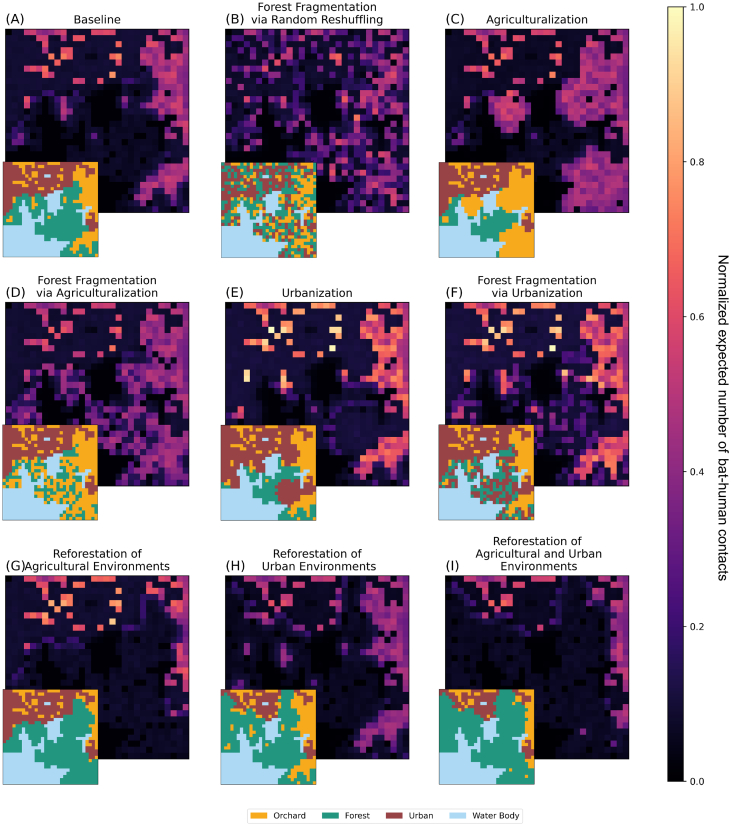
Land-use change restructures human–bat contact intensity. Panels show the normalized expected number of bat–human contacts, averaged across simulations and normalized across scenarios using min-max normalization to facilitate comparison as raw values are proportional to contact intensity rather than interpretable as absolute contact counts per unit time. Panel (A) is the baseline contact intensity, Panel (B) shows the contacts intensity at 50% habitat forest fragmentation via random reshuffling, Panels (C) and (D) show the contact intensity at 50% agriculturalization via expansion and fragmentation, respectively. Panels (E) and (F) show the contact intensity at 50% urbanization via expansion and fragmentation, respectively. Panels (G)-(I) show the contact intensity at 50% reforestation when orchards, urban areas, or both, respectively, are reforested. In the bottom left corner of each panel, we provide the corresponding landscape for reference. Created in BioRender. Stafford, E. (2025) https://BioRender.com/epdih5i

Fig. 5 shows how the LCLUC scenarios affect human–bat contacts across landscape change scenarios and, thus, helps explain why we see the changes in zoonotic spillover in Fig. 3. These patterns are shown here for the resource-poor forest condition, which generally exhibited stronger responses to landscape change across scenarios.

In Fig. 5E and F, we find that both urban expansion and forest fragmentation via urbanization increase the intensity of high-contact areas in orchards across all scenarios. This effect is driven in part by habitat loss, which forces flying foxes to concentrate in the remaining forest patches. As urban development encroaches on forest patches, the forest patches become increasingly adjacent to urban areas, creating more opportunities for human–bat interactions and amplifying the potential for spillover through landscape edge effects (see also Fig. S2). This leads to a particularly strong effect due to the scattering of urban patches within forest clusters, which increases urban–forest edge interfaces and edge-related human–bat contact. In contrast, direct urban expansion (Fig. 5E) produces fewer new interfaces, i.e., a lower degree of edge effects, resulting in a smaller increase in contact intensity.

Fig. 5C and D show that agricultural expansion and agricultural-driven forest fragmentation produce a similar overall trend, but instead of intensifying contacts, they expand the spatial extent of high-contact regions, which is not the same as edge effects. Notably, forest fragmentation in this context (Fig. 5D) reduces the intensity of contact in newly converted areas compared to agricultural expansion alone (Fig. 5C).

Over a relatively small landscape (30 × 30), forest fragmentation via random reshuffling doesn't have a great effect on how flying foxes use the landscape (assuming that the quality of resources in a patch is independent of the cluster size – number of adjacent patches of the same type), and, thus, had only a small effect on the number of patches with a high or low contact density. Figs. 5G-5I show that reforestation of both anthropogenic landscape types decreased the number of high-contact patches.

## 4 Discussion

In this study, we developed a novel individual-based model of the small-scale foraging behaviors of flying foxes, based on optimal foraging theory. We applied the individual-based model to synthetic landscapes to analyze LCLUC-induced flying-fox foraging patterns under scenarios of ecological fragmentation, agriculturalization, urbanization, and reforestation. Additionally, we evaluated the specific effects of forest degradation within these scenarios.

We found that both agriculturalization and urbanization significantly influenced small-scale flying-fox movements, whereas forest fragmentation via random reshuffling of patches had a relatively small impact. However, forest fragmentation due to urbanization produced different dynamics than direct forest loss from the expansion of urban areas. When brought about through urbanization, forest fragmentation increased edge effects—ecological interactions occurring at the boundaries between contrasting habitats—heightening the potential for human–animal contact at urban–forest interfaces and, thus, the risk of zoonotic spillover. This result suggests that prevention measures should prioritize forest expansion over the creation of green spaces within urban areas—or, alternatively, that urban green zones should implement practices to minimize human–flying fox contact. It also highlights the need for more comprehensive ecological knowledge of flying foxes in urban environments.

Our results also highlight the importance of forest degradation—represented here through resource abundance—in shaping spillover dynamics. When forests were resource poor, agriculturalization produced a stronger increase in spillover risk than when forests were resource rich, reflecting the bats' greater reliance on orchards when native resources were scarce. Likewise, reforestation was more effective at reducing spillover risk when forests were resource rich, meaning that when habitats can successfully be restored to high levels of quality (e.g., to near pristine-like), the restoration actions also more strongly reduce bat aggregation in human-dominated areas. However, in the context of urbanization, this pattern reversed beyond a threshold of landscape change: spillover risk became higher when forests were resource rich than when they were resource poor. This occurs because resource-rich forests sustain larger bat populations, and as urban areas encroach, the resulting edge effects bring more bats into closer proximity with humans, intensifying contact rates. This interaction highlights that the effects of land-use change depend critically on the ecological context—particularly the underlying resource richness of forests—and that mitigation strategies must account for how habitat quality mediates the impacts of urbanization on spillover risk.

Our simulation results align closely with empirical observations of flying-fox movements and spillover risk. For example, Eby et al. (2023) documented increased foraging in agricultural areas following a combination of habitat loss and habitat resource abundance—correlating with higher henipavirus spillover in eastern Australia—an outcome our model reproduces by showing that fragmentation and urban expansion funnel bats into resource-rich farmland, elevating contact with livestock and humans [41], and that decreased forest resource abundance had similar qualitative effects. Furthermore, our findings supply the process-based, quantitative evidence called for by Plowright et al. (2021) [10], demonstrating how specific land-use transitions create high-risk bat–human interfaces and identifying which mitigation strategies (e.g., enhanced habitat connectivity versus forest expansion) are most effective under different scenarios. Taken together, these parallels show that our work not only corroborates key field studies but also bridges a critical gap by offering a scalable, individual-based modelling framework that directly links land-use change to bat behavior and zoonotic spillover potential.

While our study offers a robust theoretical framework, it relies on small-scale empirical datasets with limited sample sizes for model validation, which may not capture the full spectrum of flying-fox behaviors. With broader ecological datasets on foraging movements and resource distributions, our framework can be calibrated to particular regions and habitats, reducing reliance on assumptions. Moreover, although our current model emphasizes short temporal and spatial scales, it can be extended to incorporate processes relevant for also longer-term population dynamics including reproduction, seasonal shifts, migratory movements, climate change impacts, and large-scale habitat fragmentation. Finally, by integrating measures of individual overall health status—given that viral shedding is closely linked to host condition—future iterations of our work could offer a more comprehensive, mechanistic understanding of zoonotic spillover dynamics.

Our model directly relates to the One Health objective of integrating human, animal, and environmental health by evaluating how changes in land-cover and land-use influence zoonotic spillover risk. The predictive capabilities of our simulations empower policymakers to identify high-risk scenarios of deforestation and forest fragmentation linked to urbanization and agricultural practices. Our findings demonstrate that forest fragmentation resulting from urbanization has distinct impacts compared to the direct and non-fragmenting expansion of urban landscapes that preserves forest continuity. In urban settings, landscape fragmentation – characterized by high spatial interspersion and edge complexity between forest and built-up areas – exacerbates spillover risk by increasing human–animal contact at urban–forest interfaces, suggesting that prevention efforts should prioritize forest expansion or implement practices in urban green spaces to reduce contact. Ultimately, this study provides a robust framework for guiding both further research directions and policy decisions on land-cover and land-use change, effectively advancing One Health strategies for preventing zoonotic spillover.

## Declaration of generative AI and AI-assisted technologies in the manuscript preparation process

During the preparation of this work the authors used ChatGPT in order to edit the article text for improved readability. After using this tool/service, the authors reviewed and edited the content as needed and take full responsibility for the content of the published article.

## CRediT authorship contribution statement

**Erin Stafford:** Writing – review & editing, Writing – original draft, Visualization, Validation, Software, Methodology, Investigation, Formal analysis, Conceptualization. **Åke Brännström:** Writing – review & editing, Supervision, Methodology, Conceptualization. **Kyrre Kausrud:** Writing – review & editing, Supervision, Methodology, Conceptualization. **Henrik Sjödin:** Writing – review & editing, Supervision, Methodology, Conceptualization.

## Declaration of competing interest

Erin Stafford reports financial support was provided by the European Union through Horizon Europe (Grant Agreement No. 101095444). Henrik Sjödin reports financial support was provided by the European Union through Horizon Europe (Grant Agreement No. 101095444). Kyrre Kausrud reports financial support was provided by the European Union through Horizon Europe (Grant Agreement No. 101095444). Henrik Sjödin reports financial support was provided by BEPREP (Grant Agreement No. 101060568). Åke Brännström declares no competing financial interests or personal relationships that could have appeared to influence the work reported in this paper.

## Data Availability

Data will be made available on request.

## Appendix A Detailed methods description

### A.1 Mathematical description of the IBM

In this section, we provide a detailed description of the mathematical model simulating the daily behaviors of flying foxes. The individual-based model (IBM) includes flying foxes and landscape patches.

We model landscape patches, which can be one of five landscape types, as the food resources available for flying foxes in that patch. At each time step, the resources *R*, in a patch *p,* at a given time *t,* are updated according to

(1) $$R_{p,t+1}=R_{p,t}+v_{p}R_{p,t}\left(1-\frac{R_{p,t}}{K_{p}}\right)-\sum_{b\in B_{p}}\phi \left(E_{b,t}\right),$$

where *ν*_*p*_ is the patch-specific resource regeneration rate, $B_{p}$ is the set of flying foxes in patch p, $E_{b,t}$ is the energy of flying fox *b* at time *t*, and *K*_p_ is the patch-specific carrying capacity for resources. The function $\phi \left(E_{b,t}\right)$ gives the resource consumption of a flying fox *b* at time *t*.

We model flying foxes by updating each individual's activity state (roosting, travelling, or foraging), location, and energy level at each time step. The energy *E* of a flying fox *b* at a given time *t* is updated according to

(2) $$E_{b,t+1}=\left\{\begin{array}{ll} E_{b,t}+\sigma \xi \phi \left(E_{b,t}\right)\exp\left(-\zeta \left(\tau ,B_{p}-1\right)\right)-\gamma_{f} & \text{if foraging in patch}\;p,\\ E_{b,t}-\gamma_{t} & \text{if travelling},\\ E_{b,t}-\gamma_{r} & \text{if roosting}, \end{array}\right)$$

where *σ* and *ξ* are the resource conversion rate and the energy gained per resource unit, respectively. The parameter *τ* is the time spent in the current patch, and $B_{p}-1$ is the number of other flying foxes in the current patch. The function $\zeta \left(\tau ,B_{p}-1\right)$ is a discount rate for staying in the same patch and is given by

(3) $$\begin{array}{c} \zeta \left(\tau ,B_{p}-1\right)=\epsilon_{t}\tau +\epsilon_{b}\left(B_{p}-1\right) \end{array}.$$

We include this term to reflect territorial behavior when foraging and the need for diet variation, whereby individuals must search for different resource types across distinct patches rather than repeatedlyy exploring a single patch. We let the resource consumption rate, $\phi \left(E_{b,t}\right)$, depend on a flying fox's energy needs such that the foraging rate increases when energy reserves are too low and decreases when reserves are too high to maintain a desired body mass. The logistic decay function used for this is

(4) $$\phi \left(E_{b,t-1}\right)=\min\left(\frac{2\phi_{M}}{1+\rho^{-\frac{E_{b,t-1}}{\;\xi }}\;},,,R_{p,t}\right),$$

where *ϕ*_M_ is the foraging rate when energy reserves are zero and 2*ϕ*_M_ is the limiting value. The base, *ρ*, is chosen such that 0 *< ρ <* 1, and ξ is the preferred level of energy reserves.

In this model, a flying fox chooses to leave to forage in a new patch when its marginal energy gain from remaining in the current patch is smaller than the net average energy gained in that patch,

(5) $$E_{b,t+1}-E_{b,t}<E_{p}^{*},$$

where the $E_{b,t+1}$ is the expected energy in the next time step if remaining in that patch. We calculate $E_{b,t+1}$ using the right-hand side of eq. (2), and the net average energy gain is given by

(6) $$\begin{array}{c} E_{p}^{*}=\frac{E_{c}-\gamma_{t}T_{p}}{T_{p}+\tau } \end{array}\;.$$

Here, *E*_*c*_ is the change in energy while foraging in the current patch and *T*_*p*_ is the time to travel to patch *p*.

When a flying fox decides to change locations while foraging, the new patch is randomly selected from the options within the maximum flight distance where eq. (5) is true. The new patch is chosen according to a probability distribution that considers available resources, distance, and patch familiarity. The probability that a patch p (out of patches P) is chosen (u) is

(7) $$\begin{array}{c} \mathbb{P}\left(u=p\right)=\frac{\max\left(10\delta \left(D_{p}\right),1\right)\cdot \max\left(V_{p},1\right)\cdot \frac{R_{p}}{\sum_{p\in P}R_{p}}}{\sum_{p\in P}\mathbb{P}\left(u=p\right)} \end{array},$$

where *δ*(*D*_*P*_) is a Kronecker delta such that *δ*(*D*_*p*_) is 1 if *D*_*p*_, the distance to patch *p,* is less than a fixed perceptual radius (assumed to be 10 km in the absence of empirical data) representing the spatial scale over which flying foxes are assumed able to gauge the available resources based on their keen sense of smell and local environmental knowledge and 0 otherwise. Moreover, *V*_*p*_ is the number of times a flying fox has visited patch *p*. Therefore, the probability of choosing a patch is higher for closer patches, and flying foxes show patch loyalty when foraging.

In addition to changing patches while foraging, flying foxes also change roosts following a similar decision model. Forest patches are randomly chosen to be roosts at the beginning of the simulation, and flying foxes are randomly assigned to roosts during initialization. Similarly to eq. (5), flying foxes will decide to change roosts if

(8) $$E_{b,t+1}-E_{b,t}<E_{r}^{*},$$

where $E_{r}^{*}$ is the net average energy gained while based in roost *r* and is given by

(9) $$\begin{array}{c} E_{r}^{*}=\frac{E_{R_{c}}-\gamma_{t}T_{r}}{T_{r}+t_{r}}. \end{array}$$

Here, *E*_*Rc*_ is the energy gained, or lost, while based in the current roost, *T*_*r*_ is the time to travel to roost *r*, and *τ*_*r*_ is the time based out of the current roost. If the decision to change roosts is made, bats will randomly choose from the other roost for which Eq. (8) is true.

### A.2 Formulation of spillover risk

We assume exponentially distributed waiting times such that the probability that at least one spillover event occurring is given by

(10) $$Z=1-\exp\left(-\mathrm{\lambda t}\right),$$

where *t* is the number of time units in the simulation when flying foxes are foraging, and *λ* is the average number of spillover events per unit time. We define *λ* as

(11) $$\begin{array}{c} \lambda =\mathrm{c\tau p}\sum_{i=1}^{L}\sum_{j=1}^{G_{i}}H_{\mathrm{ij}}B_{\mathrm{ij}}. \end{array}$$

Here, *c* is the per-capita contact between humans and flying foxes, *p* is the proportion of the flying-fox population that is infected with a pathogen, and *τ* is the probability of infection given an infectious contact.

Contacts can include direct or indirect contacts. In the summation term, we sum over the landscape types (*L*) and grid spaces of each landscape type (*G*_*i*_). The term *H*_*ij*_ is a constant that gives the number of people in grid *j* of landscape *i*. Humans are distributed among patches according to landscape type and neighboring landscape types. Moreover, we assume that all humans are susceptible to infection.

The term *B*_*ij*_ is the average number of flying foxes in grid *j* of landscape *i*. To calculate *B*_*ij*_, we use

(12) $$B_{\mathrm{ij}}=\frac{1}{t}\sum_{i=0}^{t}b_{\mathrm{ij}}\left(t\right),$$

where *b*_*ij*_(*t*) is the number of foraging flying foxes in location (*i,j*) at foraging time *t*. Then, *Z* is given by

(13) $$Z=1-\exp\left(-\mathrm{tc\tau p}\sum_{i=1}^{L}\sum_{j=1}^{G_{i}}H_{\mathrm{ij}}B_{\mathrm{ij}}\right).$$

### A.3 Model parameterization and validation

We validate our model by comparing the behavior of the modelled flying foxes with empirical observations. We recognize that flying fox behavior varies widely across studies, presumably due to differences in landscape heterogeneity and resource availability as well as differences between the taxonomic groups studied. For example, one study in central Thailand found that flying foxes predominantly foraged in agricultural landscapes [47], while research in Cambodia reported that they foraged most in residential areas [46]. In Australia, foraging site preferences have been shown to differ among non-urban, minor-urban, and major-urban areas [67]. Most of the available validation data are based on small-scale GPS tracking studies of limited numbers of flying foxes, capturing fine-scale activity patterns in both time and space. To ensure relevance, we focus on data from Southeast Asia, where bat-borne zoonoses are an increasing threat [23], [24], [25]. The behavioral metrics we aim to match include the distance travelled per night, the percentage of time spent in different habitat types, and the number of foraging trips per night.

We parameterized the model using available data on the metabolic rates and foraging behaviors of flying foxes. When possible, we relied on measurements from grey-headed flying foxes (*Pteropus poliocephalus*), which are the most comprehensively studied species [65], [66]. Many parameters remain difficult to estimate precisely because of the limited number of field studies and small sample sizes. For parameters lacking empirical estimates, we selected values that produced realistic model outputs consistent with observed flying-fox behavior in terms of foraging habitat preferences and the number of foraging sites visited per night. Table S1 in the Supplementary Information lists all parameter values used in the simulations.

Unknown parameters were calibrated manually on a synthetic test grid to ensure realistic energetic dynamics and movement behavior. Calibration targeted two criteria: (i) bats maintained stable energy levels (neither starving nor accumulating excess reserves) and (ii) individuals changed foraging patches two to three times per night on average, matching empirical observations of nightly site use by *Pteropus* species.

After calibration, the model was run on a spatial grid derived using land-cover data from the Global Land Cover product with Fine Classification System (GLC_FCS30) [69] so as to reflect the study region of Weber et al. (2015) [47]. To initialize foraging histories, individuals selected patches within their roost location, foraging range, and assumed resource availability per habitat type. Initial foraging histories were also weighted by the number of patches of each type available locally. This generated individual-specific initial foraging histories that reflected both behavioral tendencies and landscape structure.

Model outputs were compared against empirical studies describing flying-fox foraging and movement behavior. The simulated mean nightly travel distance (27 km) aligns closely with the values reported by Weber et al. (2015) [47], who found that most individuals remained within 20 km (range ≈ 8–50 km) of their roosts. After calibrating unknown parameters through a manual adjustment process to align our outputs with empirical data, our model produces a mean nightly distance of 32.0 km, with little variation among individuals. Weber et al. (2015) also reported that flying foxes foraged almost exclusively in orchards, plantations, mixed tree rows between ponds, farmyards, fallows, and tree stands [47]; similarly, flying foxes in our model of the validation-specific synthetic landscape spent the majority of their foraging time in orchards (89% of simulation time). By contrast, Schloesing et al. (2020) [46] reported that residential areas were the most frequently used foraging sites. This apparent difference likely reflects variations in land-cover classification: the “residential” category in Schloesing et al. included backyards and fruiting trees, which in our framework correspond to the *orchard* category. When this correspondence is recognized, our results align with their conclusion that flying foxes preferentially exploit productive, human-modified habitats. Furthermore, we compared the number of unique foraging searches per night to the findings of Schloesing et al. and found our model similar to with less than two foraging searches per night on average in orchard and forest settings [46].

Together, these comparisons demonstrate that the model reproduces plausible movement distances, habitat use, and nightly site switching characteristic of flying foxes across heterogeneous landscapes. This provides confidence that the framework captures key behavioral mechanisms relevant to theoretical explorations of how resource distribution and landscape change might influence spillover risk.

### A.4 Land-cover and land-use change scenario implementation

We examine the potential effects of land-cover and land-use change (LCLUC) on flying-fox behavior—and consequently on the risk of zoonotic spillover—by analyzing how different landscape modifications affect flying-fox behavior and spatiotemporal distribution of their population densities in relation to the spatial distribution of assumed human densities. The scenarios we consider include agriculturalization, urbanization, forest fragmentation (via random reshuffling, agriculturalization, and urbanization), and reforestation. Fig. 2(A)-(I) illustrates examples of these LCLUC scenarios. Forest fragmentation via random spatial reshuffling is implemented by randomly switching a proportion of forest patches with urban or orchard patches. Agriculturalization and urbanization are modelled by converting some proportion of nearby forest patches to orchard or urban patches, respectively. Fragmentation, resulting from either agriculturalization or urbanization, is implemented by randomly switching a proportion of forest patches to orchard or urban patches, respectively. Although each landscape contains the same total amount of resources, the spatial distribution of these resource amounts differs across scenarios, following the spatial organization of the respective landscape types in each realization. To explore individual LCLUC scenarios, the relative amount of resources between patch types is kept constant. For example, if orchard patches have two food-resource units for every resource unit in forest patches, this ratio will be constant across the LCLUC scenarios. However, as orchards replace forests during agriculturalization, the average amount of resources per patch in both patch types would decrease so that the total across the grid is unchanged. This decision is made so that we explore the effects of landscape structure alone on spillover risk.

## Appendix B Supplementary data

Supplementary material Image 1
