# Supplementary material for: Modelling land use-induced foraging distributions of flying foxes and emerging spillover risks
Source: One Health. 2026 Jan 14;22:101333. doi: 10.1016/j.onehlt.2026.101333 (PMC12907234; doi:10.1016/j.onehlt.2026.101333)
Supplement: Supplementary file 1 — Supplementary material [file mmc1.pdf]

Supplementary Information: Modelling land-use-induced foraging distributions of flying foxes and emerging spillover risks

Erin Stafford[a*], Åke Brännström[b,c,d], Kyrre Kausrud[e], and Henrik Sjödin[a,f]

[a]Department of Epidemiology and Global Health, Umeå University, Umeå, Sweden
[b]Department of Mathematics and Mathematical Statistics, Umeå University, Umeå, Sweden
[c]Complexity Science and Evolution Unit, Okinawa Institute of Science and Technology Graduate University (OIST), Okinawa, Japan
[d]Advancing Systems Analysis Program, International Institute for Applied Systems Analysis (IIASA), Laxenburg, Austria
[e]Norwegian Veterinary Institute, Ås, Norway
[f]Department of Wildlife, Fish and Environmental Studies, Swedish University of Agricultural Sciences, Umeå, Sweden
*Corresponding author: erin.stafford@umu.se

Supplementary Tables

Parameter	Meaning	Value	Source
N	Total bat population size across the landscape	10000	Estimated from [1,2]
σ	Resource conversion rate	0.90	Estimated from [3] using the sugar absorption rates. We assume that flying foxes have similar absorption efficiency as other fruit bat species.
ξ	Food to energy conversion rate	3.14 kJ/g	Estimated as energy per g of the average mango, , the most commonly consumed fruit according to [2].
ϵ_t	Discount term associated with time spent in the current patch	0.005	Fitted to achieve a realistic number of foraging searches per night.
ϵ_b	Discount term associated with other flying foxes in the current patch	0.005	Fitted to achieve a realistic number of foraging searches per night.
γ_f	Metabolic rate while foraging	48 kJ/hr	Assumed to be less than flying metabolic rates and greater than resting metabolic rates.
γ_t	Metabolic rate while travelling	90 kJ/hr	Estimated using findings from [4] that flying metabolic rates for bats are 15-16 times larger than resting rates.
γ_r	Metabolic rate while roosting	5.80 kJ/hr	Estimated from [5] using the BMR of *P. hypomelanus*, as it is close in size to *P. lylei*. Conversion to kJ/hr was done using standard conversion rates [5] and the average body mass of bats in [2].
$\phi_{\max}$	Y-intercept of foraging function, based on normal foraging rate of captive flying fox	30 g/hr	Estimated from [6] by assuming 12 foraging hours per day and 350g of fruit per day.
ρ	Base of the component exponential function in the foraging function	0.25	Fitted to achieve a realistic number of foraging searches per night.
E_{op}	Goal amount of energy stores	600 kJ	Estimated by assuming 3% of body mass is fat stores.
ν_p	Resource regeneration rate of patch p	0.05-0.1 g/day	Chosen randomly from a normal distribution for each patch.
$K_{f,p}$	Maximum resources of forest patch p	1000-2000 g	Chosen randomly from a normal distribution for each forest patch.
$K_{o,p}$	Maximum resources of orchard patch p	2000-4000 g	Chosen randomly from a normal distribution for each orchard patch.
$K_{u,p}$	Maximum resources of urban patch p	10-100 g	Chosen randomly from a normal distribution for each urban patch.
$K_{w,p}$	Maximum resources of water-body patch p	10-100 g	Chosen randomly from a normal distribution for each water-body patch.

Table S1. Description of parameters used in the individual-based model. Realistic values from cited sources were used when available, but many parameters were fitted or chosen for similarity to [1,2].

Supplementary Figures

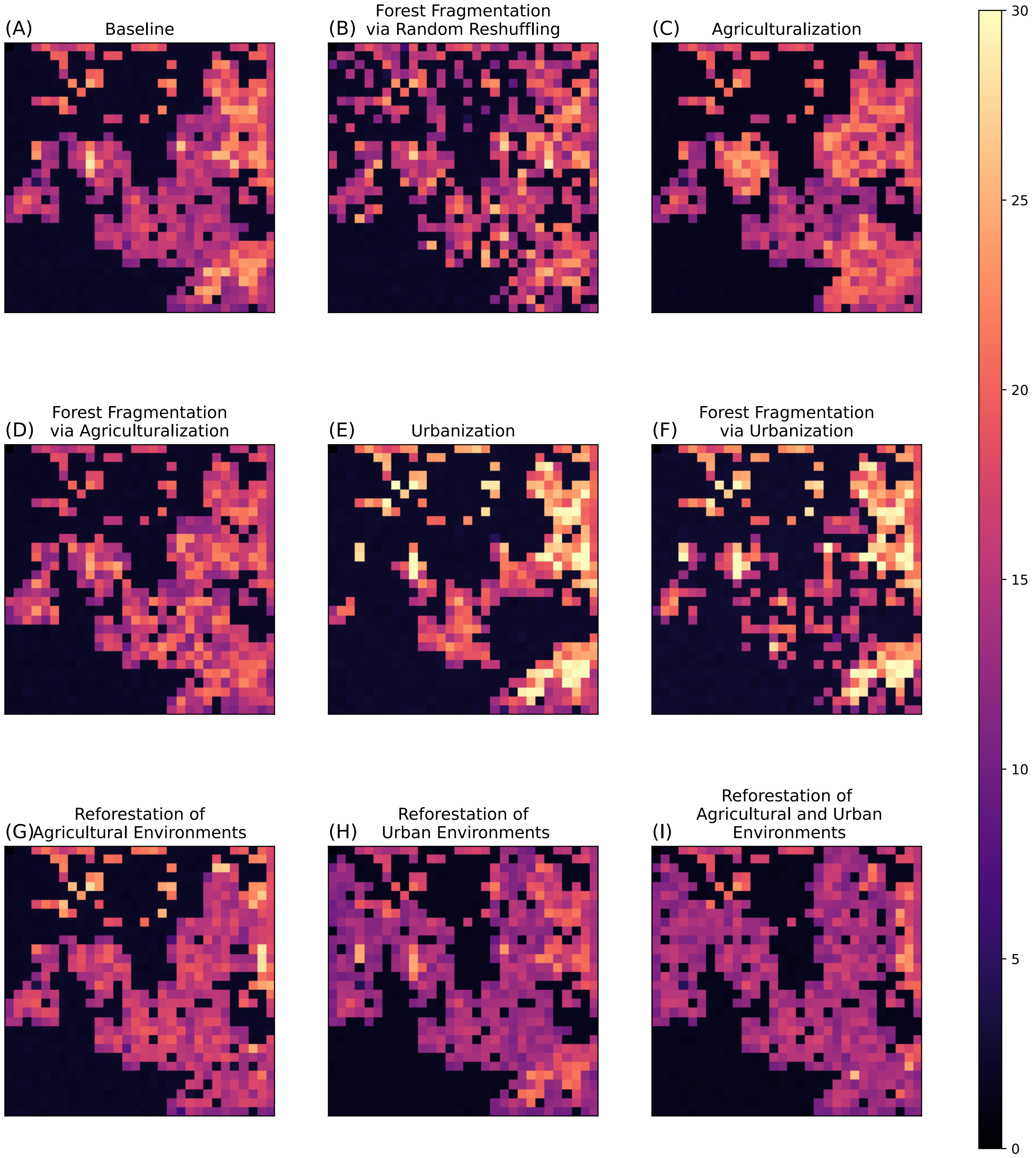

Figure S1: Visualization of behavior changes. Figure (A) is the baseline average density of flying foxes, Figure (B) shows the average density at 50% habitat forest dispersion, Figures (C) and (D) show the average density at 50% agriculturalization without and with fragmentation, and Figures (E) and (F) show the average density at 50% urbanization without and with fragmentation. Figures (G)-(I) show the effects of reforestation on flying-fox densities. Figures (G) and (H) are for 50% of orchards and 50% of urban environments reforested, respectively. Figure (I) shows the results for 50% of both urban and orchard patches reforested. The color scale, given by the color bar, represents the average number of bats per patch during foraging hours.

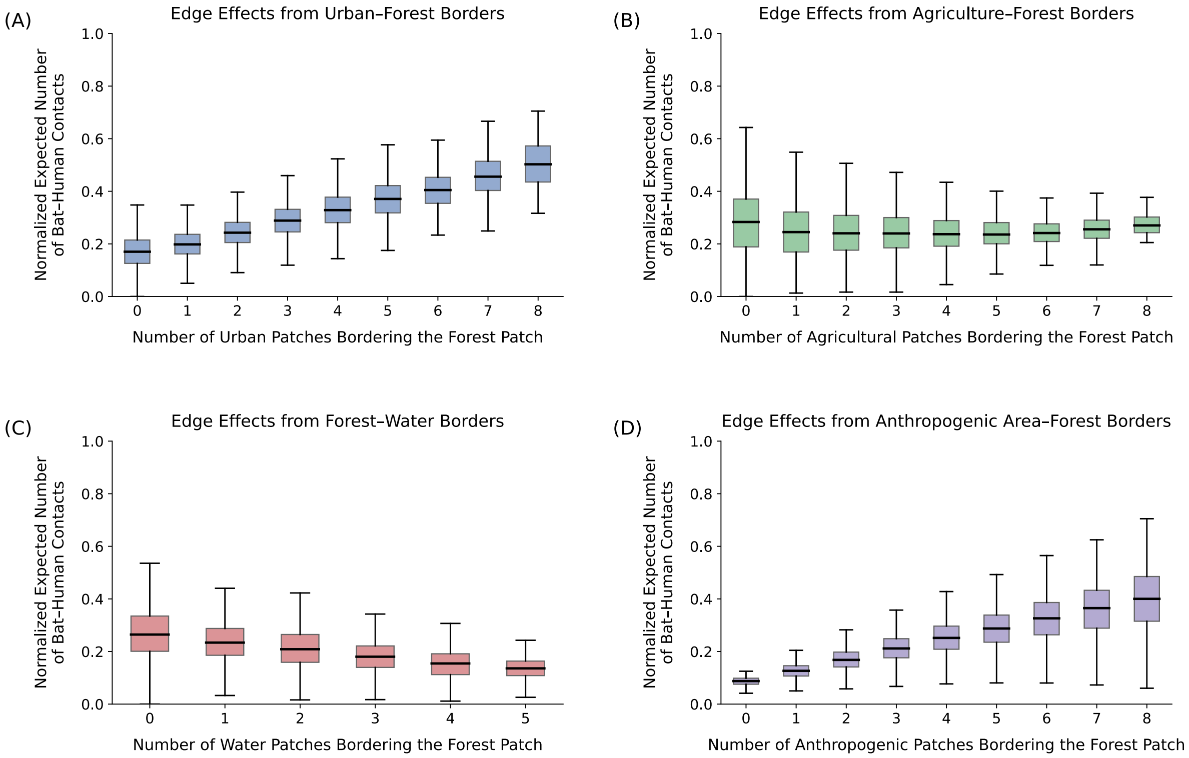

Figure S2: Edge effects at forest boundaries. Distribution across simulations of the normalized expected number of bat–human contacts in forest patches, grouped by the number of neighboring patches of different land-use types: urban (A), agricultural (B), water (C), or the combination of urban and agricultural (D), as indicated by each panel. The normalized expected number of contacts (y-axis) provides a relative measure of contact intensity within each simulation. Values are normalized using a min–max scaling across all simulations and panels, such that contact intensities range between 0 and 1. Results are shown for the scenario in which 50% of the forested landscape is altered via random reshuffling, ensuring representation of forest patches with as many non-forest neighbors as possible; only in panel (C) were we unable to cover all possible neighbor counts. We center the analysis on forest patches, as these represent the natural habitat of flying foxes and are the typical focus of discussions of edge effects. In panel (A), the normalized expected number of bat–human contacts in forest patches increases with the number of neighboring urban patches, reflecting elevated spillover risk associated with increased urban–forest boundary extent under this model configuration. In panel (B), contact intensity also generally increases with the number of agricultural neighbors; the initial decrease at low neighbor counts likely arises because forest patches with no agricultural neighbors often border urban patches, which dominate contact generation. In panel (C), contact intensity decreases as the number of neighboring water-body patches increases, consistent with the assumption that water patches contain few or no humans, reversing the edge effects observed near high-density human areas. In panel (D), urban and agricultural patches are grouped into a broader anthropogenic category, yielding patterns similar to those observed in panel (A). The shape of the relationships shown, including the near-linear form observed in some panels, depends on modeling assumptions, including the distribution of humans across landscape types, the availability of foraging resources across land-use types, and the assumption that forest-stand size does not directly affect bats' willingness to forage within a patch. Under alternative assumptions, the form of the contact distributions across neighbor counts may differ. Nonetheless, the figure illustrates how edge effects can generate increasing relative contact intensity over at least part of the range of possible neighbor configurations, particularly where areas of high human density border landscapes commonly used by foraging flying foxes.

Sources

1. E. Schloesing, R. Chambon, A. Tran, K. Choden, S. Ravon, J.H. Epstein, et al., Patterns of foraging activity and fidelity in a southeast Asian flying fox, *Mov. Ecol.* 8 (2020) 46. https://doi.org/10.1186/s40462-020-00232-8.

2. N. Weber, P. Duengkae, J. Fahr, D.K.N. Dechmann, P. Phengsakul, W. Khumbucha, et al., High-resolution GPS tracking of Lyle's flying fox between temples and orchards in central Thailand, *J. Wildl. Manag.* 79 (2015) 957–968. https://doi.org/10.1002/jwmg.904

.

3. C.R. Tracy, T.J. McWhorter, C. Korine, M.S. Wojciechowski, B. Pinshow, W.H. Karasov, Absorption of sugars in the Egyptian fruit bat (*Rousettus aegyptiacus*): a paradox explained, *J. Exp. Biol.* 210 (2007) 1726–1734. https://doi.org/10.1242/jeb.02766.

4. M.D. Thomas, Physiological ecology and energetics of bats, in: T.H. Kunz, M.B. Fenton (Eds.), *Bat Biology*, Univ. of Chicago Press, Chicago, 2004.

5. J.B.D.V. Weir, New methods for calculating metabolic rate with special reference to protein metabolism, *J. Physiol.* 109 (1949) 1–9. https://doi.org/10.1113/jphysiol.1949.sp004363.

6. Government of South Australia, Department for Environment and Water, Guidelines for the captive management of flying foxes (*Pteropus sp.)* in South Australia, Department for Environment and Water, South Australia. https://cdn.environment.sa.gov.au/environment/images/DENR-Flying-Fox-Guidelines.pdf
